# Study protocol: the pragmatic, exploratory DELTA-JU trial of the group-based multimodal DELTA intervention for abstinent adolescents with substance use disorders living in youth welfare institutions

**DOI:** 10.3389/fpsyt.2023.1025347

**Published:** 2023-05-26

**Authors:** Sören Kuitunen-Paul, Lukas A. Basedow, Veit Roessner, Yulia Golub

**Affiliations:** ^1^Department of Child and Adolescent Psychiatry, Faculty of Medicine, Technische Universität Dresden, Dresden, Germany; ^2^Chair of Clinical Psychology and Psychotherapy, Faculty of Behavioral and Social Sciences, Technische Universität Chemnitz, Chemnitz, Germany; ^3^Department of Clinical Psychology and Psychotherapy, Faculty of Psychology, Philipps-University of Marburg, Marburg, Germany

**Keywords:** addiction, clinical trial, dependence, drug abuse, recovery, therapy, treatment, waiting list control group

## Abstract

**Background:**

The DELTA intervention contains 16 weekly group sessions plus additional individual sessions and educational session for parents. It aims to reduce substance use and related problems such as substance use disorders (SUD) in adolescents. Recent results indicated positive effects in psychiatric outpatients. Conducting DELTA in youth welfare settings seems feasible, however, organizational and content adjustments such as smoking cessation elements should be added in order to reduce relapse risks and to prevent negative health consequences.

**Methods/design:**

The pre-registered DELTA-JU study (German Clinical Trials Register, DRKS00027913) is separated into three stages: In the adjustment stage during months 1–4, we will revise the DELTA manual based on semi-structured interviews (*n* = 10) with personnel from youth welfare institutions specialized in serving adolescents with SUD in the study region, analyzed with content analysis. In the sampling stage during months 5–22, participants qualifying for a SUD and willing to regularly participate in the 16 weekly DELTA-JU group sessions will be enrolled to either one of two arms (cluster randomization: immediate intervention, waitlist with subsequent intervention 16 weeks later). Adolescents will be assessed at baseline and follow-up (16 weeks after first group session) with an additional pre-assessment (16 weeks before intervention starts) for the waitlist group. Assessment procedures include questionnaires and clinical interviews among others. At the same time, institutional personnel will receive a 1-day workshop on SUD-relevant topics based on the DELTA parental education group and on feedback from the qualitative interviews. Personnel will also be assessed twice with questionnaires. In the dissemination stage during months 23–24, final study evaluation results will be prepared and submitted for publication.

**Discussion:**

This study will create a setting-specific manual for vulnerable adolescents suffering from SUDs, and, in many cases, from co-occurring mental disorders. If shown to be effective, DELTA-JU can be disseminated within other institutions of youth welfare.

## Introduction

Adolescents aged 12–18 years are likely to experiment with psychoactive substances such as alcohol and cannabis (1), with 2–10% of those who use substances developing a substance use disorder (SUD) over the course of the next years (2). SUDs are characterized as chronic mental disorders that tend to relapse into episodes of heavy substance use based on a set of biopsychosocial relapse determinants (3, 4). Such factors not only increase the risk for relapse, they are also prevalent among SUD outpatients. In our own sample of 204 outpatients (5), the majority qualified for more than one SUD (67%), had lived in single-parenthood homes (84%), repeated a class or left school prematurely (53%), or reported previous suicidal attempts (23%). Consequently, untreated SUDs are associated with delayed psychosocial development (6) and increased mortality (7, 8), thus indicating a need for prevention and treatment of SUDs.

In order to extend the existing treatment options for adolescents in Germany (9–11), we previously developed a multimodal, group-based outpatient intervention. This “Dresden multimodal therapy for adolescents with chronic substance use” (German abbreviation: DELTA) (12), consists of 16 structured weekly sessions in a group setting plus eight 1-on-1 sessions and eight additional education sessions for patients’ parents. DELTA combines recommended psychotherapeutic techniques and approaches such as motivational interviewing, contingency management, and cognitive-behavioral and systemic therapy, while also addressing mental disorders which commonly occur in addition to SUDs (5, 13, 14).

### Research directions regarding the DELTA manual

In our recent presentation of DELTA evaluation data in *N* = 146 psychiatric outpatients (including participants from youth welfare institutions), we presented first evidence that the DELTA intervention for adolescents with SUD is feasible, and shows small to medium non-significant effects in favor of DELTA regarding a reduction of both SUD severity and substance use at approximately 8 weeks after the last group session (15). Additionally, we found (small to medium) effects on the reduction of the depressive symptoms, understanding and influencing aversive emotions, and promoting prosocial behavior.

This power problem also precluded us from analyzing differences in treatment effects comparing outpatients at our outpatient unit with those who resided in a youth welfare institution and received DELTA there. Therefore, we can only assume that DELTA works the same way and leads to similar results in both settings. However, those settings differ from each other significantly. For example, in the outpatient institution, participants have to organize their commute to the group sessions while in youth welfare institutions, participants do not have to leave the building. Youth welfare institutions promote participation in group session as they schedule the group in accordance with their internal schedules, which is in contrast to the afternoon group slot in our outpatient facility which is at the same time where participants want to spend their free time with their peers. Based on these setting differences, we decided to gather additional data on DELTA in youth welfare settings where less than 33% of previously analyzed participants were treated.

#### Setting specifics in content

In our outpatient facility, adolescents may or may not yet have reached abstinence at the beginning of treatment (15). Thus, DELTA aimed at reducing substance use, achieving point abstinence, and finally achieving continuous abstinence (12). The initial sessions therefore focus on motivational techniques to reinforce commitment to achieve abstinence. In contrast, those in youth welfare institutions generally are abstinent with the start of their stay (15). In most cases they underwent inpatient detoxification before being transferred to the youth welfare institution. Thus these adolescents may already have gained SUD-related knowledge (“subjective utility”) and may already be motivated to remain abstinent rather than becoming abstinent. Not surprisingly, the evaluation study showed lowest satisfaction of adolescents with content regarding “strategies to reduce fear, to increase SUD-related subjective utility, and to reignite self-confidence in patients” (15). Therefore, it seems adequate to reframe respective sessions to fit setting-specific needs. Adolescents in these institutions live there full-time following structured routine involving psychological care, schooling, household chores, exercise, and other activities. They are under supervision continuously and are only allowed on weekends depending on their participation and commitment to institutional rules. Medical care (including psychiatric medication) is not provided by internal staff but is accessed the same ways as for other adolescents (appoints at external institutions/psychiatrists).

#### Setting specifics in stakeholder education

In the outpatient facility, adolescents present with their parents or legal guardians whom they reside with in most cases. These adults are important stakeholders in the treatment process (16, 17). Previous studies showed that parents of adolescents with SUD are both suffering from the situation but may also help to achieve treatment goals (18, 19). Thus, DELTA provided a parental education group, with eight 60-min sessions providing expert input (substances, SUD development, treatments, and family processes) as well as opportunities to reflect and discuss with other parents. In the youth welfare setting, parents do not reside with the adolescents. Although parental education settings were offered in all cooperating youth welfare institutions, the vast majority of parents generally did not attend them nor did they express interest. From a systemic perspective, institution personal plays the role of a quasi-parent in these settings. Institutional personnel may provide comparable involvement and effects in relation to the adolescent’s SUD and treatment. Thus, a revised DELTA manual should prepare similar educational sessions but for personnel. Based on feedback from the institutions during the DELTA evaluation study, such sessions cannot be attended weekly. Instead, they specifically asked for a one-day workshop to be held. Based on material for the parental education sessions a preliminary version for this workshop has been conceived, but it still requires modification based on personnel feedback.

#### Tackling tobacco smoking

Regarding the main intervention effect, i.e., reductions of use for most substances including alcohol and methamphetamine, nicotine use was an exception as it did not decrease during the follow-up period (15). As the only substance, nicotine use is partly accepted by the institutional house rules, and adolescents are permitted to smoke outside. Smoking tobacco to obtain nicotine is the most prevalent use form among our sample, with 88% of them reporting to have used tobacco in the past year on an average of 26.5 days per month (5). Tobacco use in adolescents often already starts in early adolescence (5), and lingers on although tobacco is not allowed to be purchased for persons younger than 18 years. Not only is smoking tobacco associated with detrimental consequences to health and quality of life (20), it is also regarded as a risk factor for relapse in adolescents who have reached abstinence from illicit substance use (21). Therefore, a revised DELTA manual should also focus on smoking tobacco by providing guideline-oriented aids (22).

### Study objectives/aims

In the publicly funded study DELTA-JU study (abbreviation of the German study title “DELTA in Jugendwohneinrichtungen”), we will aim: (A) to adjust sessions to the youth welfare institution setting and to needs of institutional personnel; (B) to evaluate whether the revised DELTA-JU manual is rated as acceptable by adolescent participants and institutional personnel; (C) to compliment previous results on DELTA effects, i.e., show that DELTA-JU is associated with reduced substance use (including tobacco use) and reduced SUD problems at FU.

## Methods and analysis

### Study design

**Figure 1:**
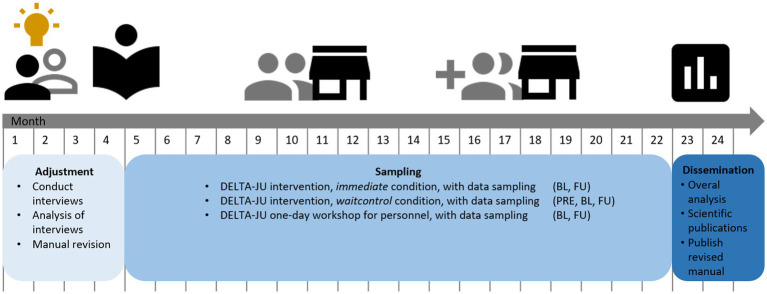
Overall study schedule and timeline.

Depending on their assigned condition, participants will be part of the trial for 17 or 33 weeks in total, see Table 1 for the SPIRIT schedule of enrollment, interventions, and assessments (Figure 1).

**Table 1:** SPIRIT Schedule of enrolment, interventions, and assessments.

TIMEPOINT	Study period					
	Enrollment	Allocation	Post-allocation	FU (immediate condition) BL (WL condition)	Post-allocation	FU (WL condition only)
	Week 0	Week 0	Week 1–16	Week 17	Week 17–32	Week 33
Enrolment						
Eligibility screen	X					
Informed consent	X					
Allocation		X				
Interventions						
DELTA-JU, immediate condition			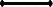			
DELTA-JU, WL condition					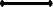	
Assessments						
Evaluation of intervention			X	X	X	X
Substance use and SUD	X			X		X
Co-occurring psychiatric problems	X			X		X
Risk factors for substance use and SUDs	X			X		X
Cognitive functioning	X			X		X
Biological markers	X			X		X

BL, baseline; FU, follow up; SUD, substance use disorders; and WL, waitlist control.

**Figure 2:**
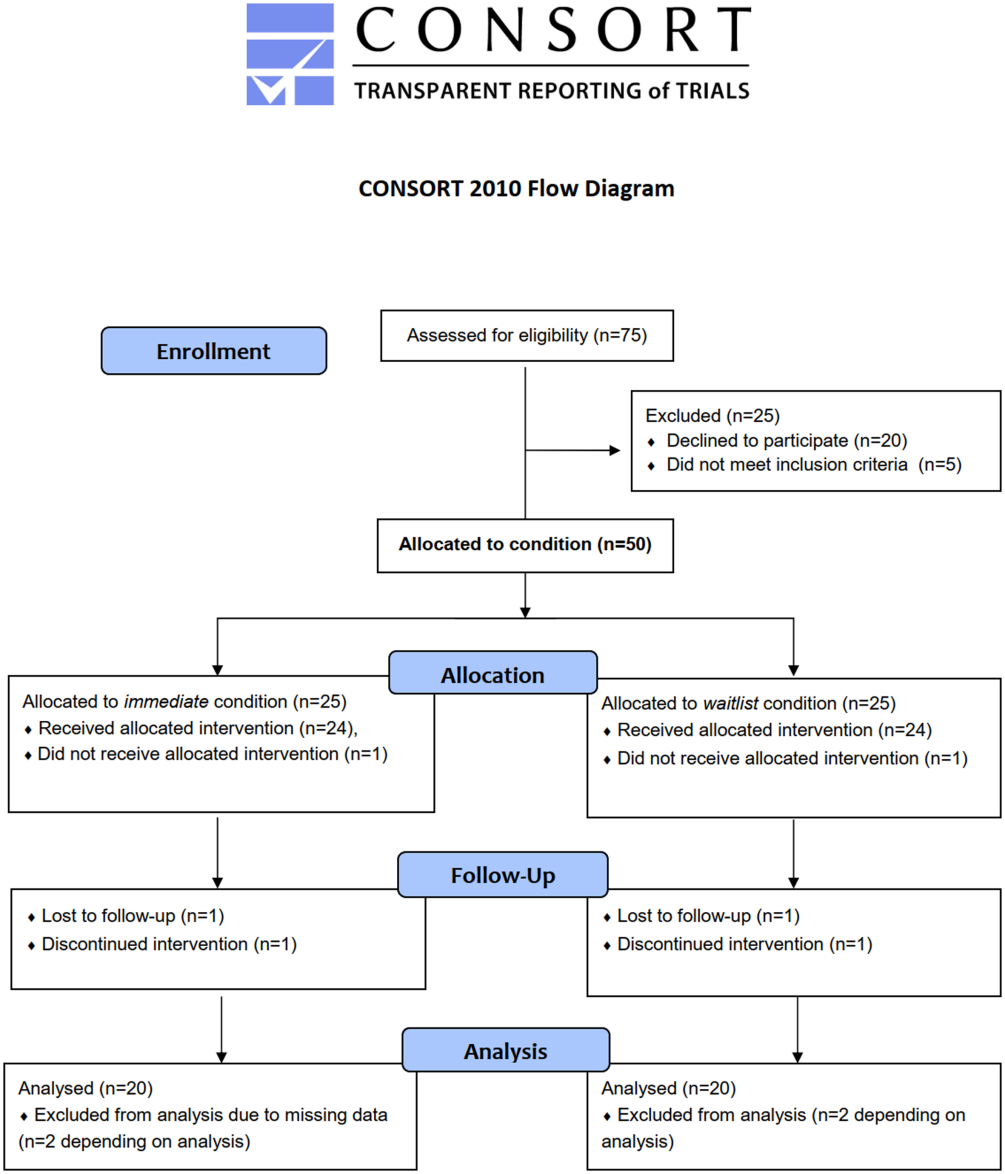
Flow diagram of anticipated allocation of adolescent participants. Note that sample sizes for allocation (*N* =  50 overall) and analysis (*N* =  40 overall) are estimated *a priori*, respectively, see section “Estimation of samples size”.

To achieve the outlined aims, we conceptualized an explanatory three-stage study over the course of 24 months, see Figure 2 for the 24-month study schedule. After adjusting the DELTA manual (12), to feedback from youth welfare institution personnel (content analysis of semi-structured qualitative interviews) in the adjustment stage, participants are cluster-randomized (institution as cluster, allocation by unblinded principal investigator who assigns the group consecutively to the sequence “intervention-waitlist-intervention-waitlist”) to either one of two study arms [immediate treatment condition vs. waiting list control group condition (WL) with SUD treatment after 16 weeks] during the sampling stage. In terms of the Medical Research Council Framework for the evaluation of complex interventions (23), this design can be considered a pragmatic trial in “phase 2,” which implies that theoretically relevant aspects have been previously identified (pre-clinical research, phase 0) (12), and that components of interventional procedures are readily available (modeling research, phase 1) (12). Further elements of a pragmatic trial are a simplified analysis design and uncontrolled environments (24). Blinding of participants, care providers, outcome assessors, or analysis is not feasible in this trial. In sum, the chosen design significantly differs in several relevant aspects from ‘classical’ cluster-randomized RCT (phase 3).

Also during the sampling stage, youth welfare institution personnel receives a newly designed one-day workshop on SUD-relevant topics. After completion of data sampling from adolescent DELTA-JU participants and personnel who evaluate the workshop, the dissemination stage starts where the DELTA-JU manual and overall evaluation results on the resulted DELTA-JU intervention are published. All measures and procedures are approved by ethics committee of the Technische Universität Dresden as an amendment to the DELTA evaluation study (approval in Jan 2022, number: EK 66022018).

#### Adjustment stage

During the adjustment stage in months 1–4, youth welfare institutions focusing on full-time housing for adolescents with SUDs are identified by contacting communal youth service authorities that typically provide long-term accommodation, in-house schooling, and psychosocial support for abstinent adolescents with SUDs. Identified institutions will receive letters or emails where study aims, procedures, and conditions are explained, asking for a collaboration. A formal cooperation agreement will be obtained with them. Institutional personnel will ask all adolescents and their legal guardians about their interest to participate, and will help to disseminate study information material. Furthermore, institutional personnel will be asked to participate in individual interviews (*n* = 10). The audio-taped interviews will take place in a separate room on an individual location to provide privacy. Interviews follow an semi-structured guideline in accordance with standards in the field of qualitative methods (25–29), with questions about the following areas of interest: (1) what are organizational challenges to implementing weekly group session in the institution (e.g., already existing therapeutic offers), (2) what session would be necessary and what problems do adolescents encounter in the institution, and (3) which needs does the personnel have regarding the one-day workshop? Responses will be analyzed with content analysis (29, 30) and/or Grounded Theory (29, 31). Thereby identified aspects in each of the three areas will be discussed by the study team so that for each aspect, at least one way how to implement it into the revision of DELTA is defined and subsequently carried out. Additionally, expert knowledge of the principal investigators will help to revise content and procedures of the manual in order to optimize its fit for the youth welfare institutional setting. The adjustment stage ends with the finally revised DELTA-JU manual including revised materials for adolescents as well as for personnel regarding the one-day workshop.

#### Sampling stage

During the sampling stage in months 5–22, adolescents will be recruited in cooperating youth welfare institutions in the study region, followed by a group-wise cluster-randomization. Adolescent participants will be prospectively assessed both at baseline (BL, a week before the intervention starts) and at follow-up (FU, 16 weeks after first group session). Those in the WL group will receive an additional pre-assessment (PRE, 16 weeks before intervention starts) for the WL to control for natural development. Assessments will take place in the youth welfare institution if possible, or in our outpatient clinic, e.g., for physical examinations. Questionnaires will be filled out in participant’s free time. Participants will be reimbursed at PRE (15 EUR), BL (20 EUR), and FU (40 EUR). The DELTA-JU group and individual sessions will be carried out as outlined below (“Interventions”) with possible changes due to the revision process. Study team members (i.e., psychologists, medical doctors, doctoral students, and student assistants from psychology, medicine, social work, or similar) who conduct the sessions or any assessment will be trained and supervised by the principal investigators.

**Figure 3:**
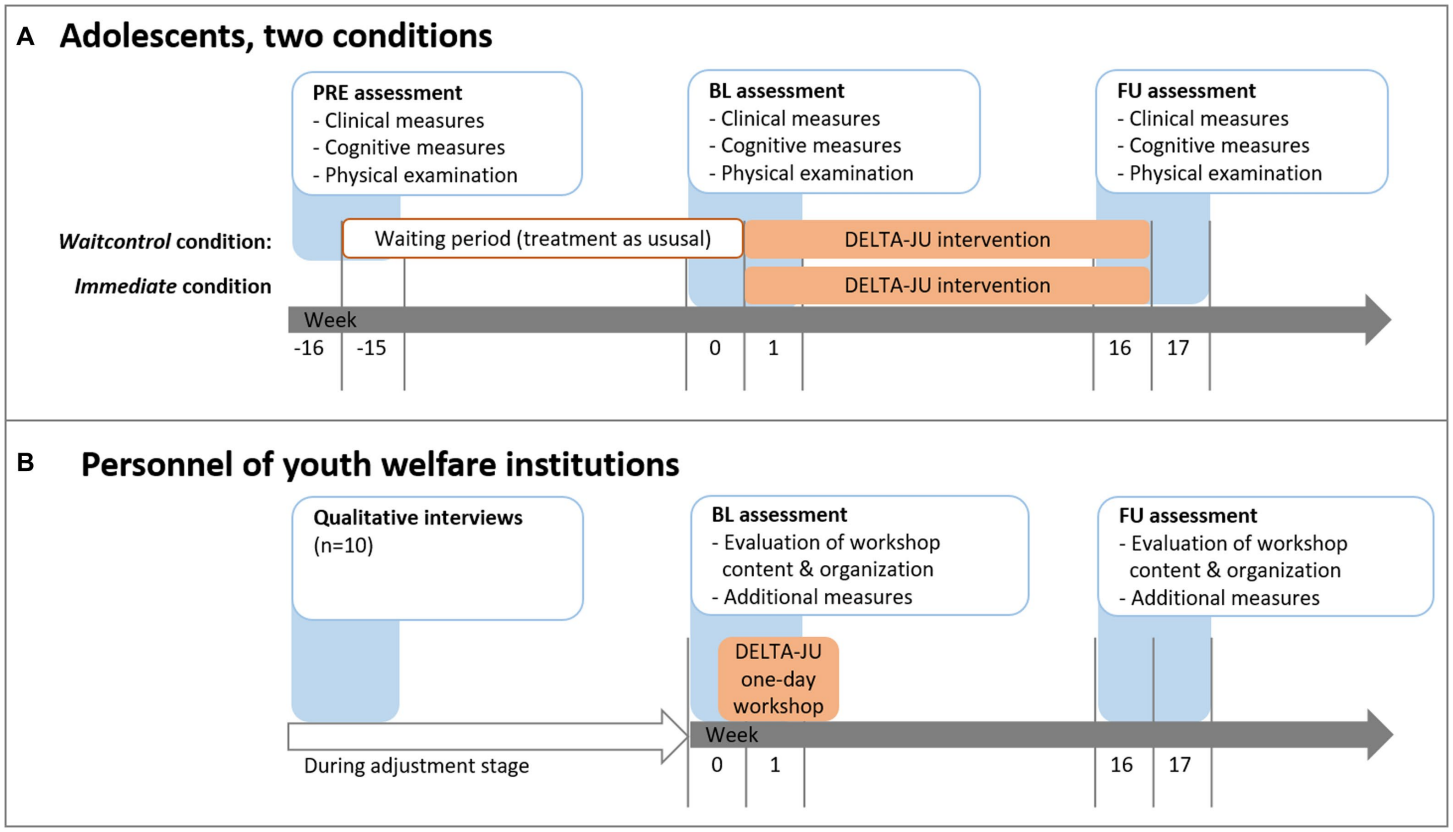
Treatment and measurement schedule for (a) adolescents and (b) youth welfare institution personnel. Timing of the 1-day workshop may vary for each institution based on time constraints of the personnel.

During the same time, personnel from the youth welfare institutions will receive the revised one-day workshop on SUD-relevant topics. Workshops will be carried out either in the institutions themselves, in our outpatient clinic, in a public place, or via video conference if necessary due to restrictions for the prevention of COVID-19 infections. Whenever possible, personnel from similar institutions will be allowed to participate in the workshop. Personnel will also be assessed twice (BL at beginning and end of the workshop day, FU 16 weeks later) with questionnaires regarding the evaluation of workshop content and organization. All procedures were conducted in accordance with the Declaration of Helsinki, publicly pre-registered and approved by the local Institutional Review Board (see “Ethics” section below). For details on the dissemination stage, see "Dissemination” section below. The treatment and measurement schedule is shown in Figure 3.

#### Setting and participants

Youth welfare institutions will be approached if they are in the study region (district of Saxony and, if feasible, surrounding areas in Brandenburg and Thuringia). Generally, those institutions specialized in SUD care are placed outside of metropolitan areas (32) in order to reduce risk factors for relapse (such as contact to drug dealers) (33). Adolescents and their legal guardians will be approached by personnel of participating institutions. If interested, study personnel will hand out information material and will answer all questions in person, via telephone or via e-mail. Consent will be given after additional verbal information to adolescents. Inclusion criteria for adolescents are: (1) aged 12.00–17.99 years, (2) SUD diagnosis, or (3) chronic substance use during the past. The only exclusion criterion is low cognitive functioning (intelligence quotient <70). For BL-FU analyses, we will not analyze participants who did not attend any of the 16 sessions for whatever reason or if study participation was discontinued due to an adverse event. Participants choosing to discontinue are immediately asked whether they want to provide data on primary outcomes anyhow. Personnel of youth welfare institutions is included if they work with at least 20 h/week in a cooperating institution. The anticipated flow of adolescent participants is shown in Figure 2. At the time of writing this protocol, study registration is completed (01. Feb 2022) and the first adolescent participants are being recruited, randomized, and enrolled (starting 02. Feb 2022).

### Interventions

#### DELTA intervention before revision

The manualized DELTA intervention (12) aims to reduce substance use and SUD-related problems to obtain abstinence and general well-being. It combines motivational interviewing, contingency management, cognitive-behavioral, and systemic therapy, see Table 2. Weekly group sessions of 60-min with 3–8 adolescents are led by up to two psychologists, who also conduct up to eight individualized 1-on-1 sessions (30 min) for each participant. Group sessions follow a structured plan including recurring elements (past-week craving review, checking homework, setting session goals, session-specific content, getting new homework, mindfulness exercise or reflection and feedback, and sporadic urine testing) and session-specific work sheets, but it also includes role plays, presentations, white-board actions, written self-evaluations, experimentation with skills boxes, mindfulness exercises etc. Attendance of all sessions is required and formally accepted within a signed “therapy contract.” Exemptions from sessions need an *a-priori* explanation (e.g., doctor’s appointment). When two or more meetings were missed without a valid explanation, or a sporadic drug urine test was positive on two occasions, adolescents have to be temporally excluded from group sessions for 8 weeks. Re-entry into the group sessions was possible after 8 weeks or a consultation with the attending therapist.

**Table 2:** Intervention content of the DELTA intervention before revision.

Session	Topic	Goal(s)
Adolescent patients group		
1.	Use and motivation to stop	Link substance use to SUDs Discover personal abstinence motivation
2.	Triggers	Identify triggers for craving and relapse
3.	Skills	Explore anti-craving skills
4.	New challenges	Develop situational anti-relapse strategies
5.	Relapse and stress	Reduce stress to prevent relapses
6.	Self-esteem	Develop self-esteem
7.	Honesty	Develop honesty in substance use self-reports
8.	Depression	Prevent and cope with depressiveness
9.	Boredom	Prevent and cope with boredom
10.	Understanding emotions	Identify emotions and associated reactions
11.	Coping with emotions	Promote activities to evoke positive emotions Avoid activities/situations associated with negative emotions
12.	Relapse justifications	Avoid situations with relapse risk
13.	Anti-relapse training	Revisit relapse prevention strategies, especially “3. Skills”
14.	Setting personal limits	Respect needs and limits of self and others learn to say “yes” and “no” if adequate
15.	Alcohol	Understand alcohol as a risky drug
16.	Review (*or optional: s*hifting addictions)	Evaluate own progress understand that excessive use of other substances or media or internet puts oneself at risk for future disorders
Parental education group		
1.	SUDs^a^	Understand SUD development
2.	Substances^a^	Understand effects of different psychoactive substances
3.	Recovering families^a^	Understand SUD treatment stages
4.	Relapse^b^	Accept relapse as possible part of recovery Develop strategies when relapse occurs
5.	Living with addiction^b^	Reflect child–parent relationship reflect own parental style
6.	Communication^b^	Reflect own communication behavior Develop alternative strategies for problematic communications
7.	Nonviolent communication^b^	Develop and train basic principles of nonviolent communication
8.	Self-care^b,c^	Understand necessity to take care of oneself Reflect on other family members/siblings

SUD, substance use disorders. ^a^based on a digital presentation; ^b^based on work sheets and material for multi-parent exchange; and ^c^an optional ninth session with individual topics may be offered.

#### Waiting list control group

Participants whose group was cluster-randomized to the WL condition are not offered group sessions until 16 weeks have passed. During this period, adolescents may seek any kind of treatment as usual, including medication for co-occurring disorders or any kind of SUD treatment. Afterwards, DELTA-JU is provided in the same way as in the immediate treatment condition described above. The pre-treatment waiting period acts as a naturalistic comparator to the treatment. It is a suitable comparator given that many of the possible confounders (housing, timespan since previous detoxification treatment, availability of concurrent treatments/medication, psychosocial support, schooling situation etc.) should be equally present or absent in both conditions. Since concomitant treatments are allowed and documented in both conditions, we will be able to control for this confounder while at the same time providing a comparator group that represents the heterogeneity of SUD care in this population.

#### One-day workshop for personnel

The workshop will be based on content both from the parental education group (see Table 2) and previous presentations held for personnel of county administrations, medical students, police officers, teachers, and educators. If applicable, we may use computerized presentations, videos, worksheets, role play instructions, case vignettes, panel discussions etc. as educational methods.

### Quantitative measurements

Primary outcomes in terms of study aims for adolescents are SUD-related subjective utility (GEJ), substance-related craving as a measure of relapse risk (MaCS), SUD severity (AUDIT, DUDIT, and FTND), and self-reported past-month tobacco use (substance use interview). For personnel, primary outcomes are SUD-related subjective utility and satisfaction ratings concerning the workshop organization (GEB-K, GEB-S). Secondary outcomes for adolescent participants are similar to those from the DELTA evaluation study (15) and include psychopathologies (BDI-II, PQ-16, UCLA-PTSD, and YSR), life satisfaction (SWLS), and perceived stress (ERI-S-10, PSS-10). For sample characterization, check of inclusion criteria, and for exploratory analysis in combination with data from that study, we will also apply additional measures that are either clinical standard in our outpatient clinic (e.g., tests of intelligence or cognitive performances) or that are of research interest yet not related to the main study aims (e.g., analysis of epigenetics in blood, cortisol in saliva and hair). Table 3 lists all quantitative measures which are applied in the DELTA-JU study to obtain outcomes. Measures related to study hypotheses (AUDIT, DUDIT, FTND, MaCS, GEJ, GEB, and substance use interview), exploratory hypotheses (BDI-II, ERI-S-10, PQ-16, PSS-10, SWLS, UCLA-PTSD, and YSR), study inclusion (MINI-KID, C-DIPS) as well as measures which were generated (GEJ, GEB) or adapted (MQ-RS, CO in breath, physical examination) for this study are presented in more detail with references to their German versions. It should be noted that items from several instruments had to be reworded so that the formal German addressing of participants (“Sie,” “Ihr”) is replaced with the informal German addressing (“Du,” “Dein”) that is more adequate when addressing children and adolescents. Details for other instruments and procedures can be found in the referenced sources.

**Table 3:** Assessment instruments for adolescent participants as well as personnel of youth welfare institutions (highlighted with *).

Construct	Reference	Instrument used per assessment		
		PRE (week −16)	Baseline (week 0)	FU (week 16)
Domain: Evaluation of intervention				
Increase in subjective utility, skills	(15)	–	–	GEJ
Increase in subjective utility, skills	–			GEB-K*
Satisfaction with one-day workshop	–	–	–	GEB-S *
Satisfaction with DELTA-JU group session (after each session)	(34)	–	GTS-P	–
Satisfaction with DELTA-JU group session (after each session)	(34)	–	GTS-T^a^	–
Domain: Substance use and SUD				
Substance use (generic interview)	(5)	interview	interview	interview
Diagnosis of SUD, co-occurring mental disorders	(35, 61, 62)	C-DIPS/ MINI-KID	C-DIPS/ MINI-KID	C-DIPS/ MINI-KID
Severity of SUD: Alcohol use disorder	(36)	AUDIT	AUDIT	AUDIT
Severity of SUD: SUDs for illicit substances	(37)	DUDIT	DUDIT	DUDIT
Severity of SUD: Tobacco use disorder	(22)	FTND	FTND	FTND
Craving for substance use	(38)	MaCS	MaCS	MaCS
Domain: Co-occurring psychiatric problems				
Depressiveness	(39)	BDI-II	BDI-II	BDI-II
Psychotic symptoms	(40)	PQ-16	PQ-16	PQ-16
Psychopathologies	(58)	YSR	YSR	YSR
Satisfaction with life	(41)	SWLS	SWLS	SWLS
Traumatic events and post-traumatic problems	(42)	UCLA PTSD	UCLA PTSD	UCLA PTSD
Perceived stress	(43)	PSS-10	PSS-10	PSS-10
Perceived stress	(44)	–	ERI-S 10*	ERI-S 10*
Domain: Risk factors for substance use and SUDs				
Tobacco use: motives	(45) ^c^	MQ-RS	MQ-RS	MQ-RS
Tobacco use: expectancies	(46) ^c^	SEQ	SEQ	SEQ
Tobacco abstinence: self-efficacy	(47)	SER	SER	SER
Tobacco abstinence: percentage of smoking peers	(48)	PSP	PSP	PSP
Temperament	(49)	SURPS	SURPS	SURPS
Sociodemographics (generic questionnaire)	(5)	–	Generic ^b^	–
	–	–	Generic*	–
Domain: Cognitive functioning				
Memory	(50)	VLMT	VLMT	VLMT
Alertness, divided attention, inhibition/impulsivity	(51)	TAP	TAP	TAP
General executive functioning	(52)	Stroop	Stroop	Stroop
	(52)	Stop-signal	Stop-signal	Stop-signal
Intelligence	(53, 54)	–	WISC-V / WAIS-IV ^d^	–
Domain: Biological markers of substance use and stress				
Carbon monoxide in exhaled breath	(22)	CO in air	CO in air	CO in air
Genome wide DNA methylation	(55)	Blood	Blood	Blood
Cortisol Cortisol	(56) (56)	Saliva Hair	Saliva Hair	Saliva Hair
Weight, height, acute physical diseases	–	examination	examination	examination
Pubertal development (questionnaire)	(57)	PDS	PDS	PDS

* self-report of personnel. ^a^ group of adolescents is rated by the therapist. ^b^ individual adolescent is rated by legal guardian. ^c^ translated or adapted for this study. ^d^ WISC until age 16, WAIS for older adolescents.

#### Severity of SUDs

Severity of SUDs is measured via three total scores of self-report instruments assessing substance use parameters as well as related problems. The Fagerström Test for Nicotine Dependence (FTND) (22) has six items with 2–4 answer options per item, resulting in a total score of 0–10 points with higher points indicating stronger problems due to tobacco use in the past week. The Alcohol Use Disorders Identification Test (AUDIT) (36) has 10 items with 3–5 answer options resulting in a total score of 0–40 points for alcohol-related problems in the past 12 months. The Drug Use Disorders Identification Test (DUDIT) (37) has 11 items with 3–5 answer options, resulting in a total score of 0–44 points for drug problems in the past 12 months.

#### Craving

The Mannheimer Craving Scale (MaCS) (38) is a self-report questionnaire with 12 items, assessing substance-related urges to consume psychoactive substances/drugs. Items are based on a five-point scale with item-specific verbatim for each point, e.g., item 11: “How strong is your urge to take the substance? 0 = I feel no urge, 1 = I feel some urge, 2 = I feel a strong urge, 3 = I feel a very strong urge, 4 = The urge is absolutely overwhelming and cannot be influenced.”

#### Substance use

Substance use is assessed via structured interview (5) by a clinical psychologist who asks for the number of use days and the amount per use day on average for each of the following substances both for past month and past year: nicotine, alcohol, cannabis, methylenedioxymethamphetamine (MDMA), amphetamine, methamphetamine, hallucinogens, opiates, inhalants, or other. These quantity and frequency reports are multiplied to obtain a quantity-frequency index (e.g., for alcohol: 10 drinking days past month × 4 standard drinks per drinking occasion = 40 consumption units per month during the past year). Additionally at BL, the interviewer will ask the same questions for past year-use as well as for age at first use per substance. Changes in the past-month quantity-frequency index for tobacco use will be a primary outcome. Other substances are only explored given that youth welfare institutions do not permit any other substance use than smoking.

#### Subjective utility

For adolescents, a self-designed questionnaire (“Gruppenevalution Jugendliche,” GEJ) from the DELTA evaluation study (15) will be used. The questionnaire contains 20 items that are related to SUD-specific subjective utility as trained in the group sessions (e.g., “I recognize my triggers,” “I’ve learned to deal with boredom,” “I have more control over my SUD,” and “I have more drug knowledge”). Items are rated on a five-point Likert scale (0 = does not apply at all, 1 = applies a bit, 2 = applies rather than not, 3 = applies most of the time, and 4 = applies always) to indicate how much participants approve each statement.

For personnel, a comparable self-designed questionnaire (“Gruppenevalution Betreuer,” GEB) was designed. One part asks for changes in subjective utility (GEB-K) induced by the one-day workshop, e.g., “I feel that I have learned new skills for coping with the child,” “I feel less alone with the substance use problems of the child,” “I feel I have gained more control over the current substance use problems of the child.” Personnel is instructed to rate it is approval for each of the 14 GEB-K statements using the same five-point Likert scale as the GEJ. The second part asks for the satisfaction (GEB-S) with the workshop, separately for organizational aspects (eight items, five-point Likert-scale from 1 = does not apply at all to 5 = absolutely applies, e.g., “The trainer was well-prepared,” “Material was well-designed and could be readily used”) and for applicability of workshop content to their daily work (six items, five-point Likert-scale from 1 = not helpful at all to 5 = very helpful, e.g., “Comorbid disorders,” “Stages of recovery”). GEB-K and GEB-S have been used during the piloting of the DELTA evaluation study (15).

#### Secondary outcomes

Depressiveness is assessed with the Beck Depression Inventory II (BDI-II) (39), a self-report questionnaire with 21 items (Likert scale ranging from 0 to 3) resulting in a sum score.

The Youth Self Report form (YSR) (58) covers a range of different psychopathologies via 118 items (Likert scale ranging from 0 to 2). We will analyze the subscales for depression-related affective symptoms (“YSR anxious/depressive”), social impairments (“YSR social withdrawal”), attention-deficit disorder-related problems (“YSR attention”), and conduct disorder-related problems (“YSR aggressive” as well as “YSR dissocial”).

Psychopathologies related to post-trauma and post-traumatic stress (PTSD) are assessed via the UCLA PTSD scale (59) that assesses all DSM-5 PTSD symptoms related to the three scales “intrusion,” “avoidance,” and “hyperarousal” (scored here as present or absent).

Psychopathologies related to prodromal symptoms of psychoticism are assessed via the 16-item Prodromal Questionnaire (PQ-16) (60), where binary-rated items are summed up to a score.

Current life satisfaction is rated by adolescents on the Satisfaction with Life Scale, German version (SWLS) (41), a five-item questionnaire. The SWLS covers global life satisfaction in contrast to related constructs such as positive affect or loneliness by asking for life conditions, achievements etc. to be rated on a seven-point Likert scale (1 strongly disagree to 7 strongly agree). Sum scores may range from 7 to 49, with higher scores indicating higher life satisfaction.

Perceived past-month stress in adolescents is assessed using the Perceived Stress Scale (PSS-10) (43) with 10 items rated on a five-point scale and resulting in a total sum score. Perceived current stress due to occupational demands are assessed via the 10-item Effort-Reward Imbalance at work scale short form (ERI-S-10) (44) with Likert-scaled items (range 1–4) and a total sum score.

#### Additional measures

Current DSM-5 diagnoses for SUDs as well as co-occurring mental disorders are assessed with the Mini-International Neuropsychiatric Interview for Children and Adolescents (MINI-KID) (35) or the Childrens’ Diagnostic Interview for Mental Disorders (C-DIPS) (61, 62) depending on license availability. Both instruments are structured interviews for DSM-5 disorders in children and adolescents with substantial to almost perfect interrater and test–retest reliability (35, 61).

Sociodemographic characteristics of adolescents are acquired via questionnaire from legal guardians, including information on participant’s age (in years), gender, parental school education (list of school graduation options), and relationship status of biological parents. Sociodemographic characteristics of personnel is obtained via standardized questions regarding age (in years), gender, school education (list of school graduation options), and current SUD-related subjective utility (percentage, from 0 = ‘none at all’ to 100 = ‘all you must know’).

Adherence to therapy is operationalized through the number of sessions attended by each adolescent (ranging from 1 to 16 sessions, with more sessions indicating stronger adherence) and by subjective rating of active participation by each adolescent itself (five-item questionnaire, GTS-P) (34). Although participants are instructed to attend to all 16 weekly sessions as well as individual sessions, thus only participants with one or more attended sessions will be analyzed. The study team member who conducts a group session will additionally rate how well the group participated actively in each respective session, and whether any adverse events occurred (nine-item questionnaire, GTS-T) (34). Drop-out from the study as well as discharge of adolescents from an institution will be recorded by study personnel.

Substance use motives specifically for smoking are assessed via the Motives Questionnaire Revised for Smoking (MQ-RS), an instrument we developed. The MQ-RS uses the 20 items and the five-point scale [ranging from 1 = (almost) never to 5 = (almost) always] from the established drinking motives measure of Cooper in its revised form (45). In contrast to drinking motive measure, we had to reword items 8 (“…about not smoking” instead of “…about not drinking”) and 10 (“…to feel the kick” instead of “…to get high”) in order to avoid alcohol-specific or smoking-unrelated wording.

Using a breath analyzing device, we will measure the amount of carbon monoxide (CO) in breath in accordance to standards in the field (63), with CO ≤ 20 ppm indicating point abstinence from tobacco smoking (22).

The physical examination is carried out by medical students who is supervised by a principal investigator. Participants are measured (height in cm, weight in kg) and examined according to clinical routines. Any deviations from normal physical development are noted in a protocol, e.g., visible wounds, signs of diseases, or basic neurological problems. Adolescents will be asked for current infections, diseases, and medications. In case of psychoactive medication or acute infectious diseases, saliva and blood will not be collected or analyzed, as these factors a confounding issues.

### Data management

Data quality is checked biannually via interim analysis and auditing by the principal investigators regarding outliers, subgroup means, and missing data patterns of primary and secondary outcome data, with the final decision to terminate the trial if applicable. As in the previous DELTA evaluation study, missing item values will be replaced by the mean of questionnaire items, if 80% or more of the items were answered (15). Items that may not be replaceable have been anticipated to appear in approx. 10% of the cases. The sample size estimation accounts for this by loss, see Figure 2.

### Data analysis

Analyses will be conducted with the most recent version of the software “IBM SPSS Statistics,” currently version 27.0. Adolescents are analyzed as randomized if they participated in at least one DELTA session (DELTA condition only). To test all non-descriptive hypotheses (see below, hypotheses 2–4), a repeated measures MANOVA will be conducted using time as factor (“within factor”) given the explanatory nature of the trial and the non-importance of between-group effects. A significant reduction over time (BL vs. FU) in all *N* = 40 participants across all metric outcomes (MaCS score, FTND score, DUDIT score, AUDIT score, and cigarettes per day during the past month according to substance use interview) indicates a relevant intervention effect. This multivariate approach will limit the chance for false-positive results as otherwise possible due to multiple testing. Effect sizes will be classified into small effects (ƞ^2^ ≥ 0.01), medium effects (ƞ^2^ ≥ 0.06), and large effects (ƞ^2^ ≥ 0.14). In case of severe non-normality of outcome variables, we will have to apply non-parametric tests instead of the repeated measures MANOVA.

#### Hypotheses

Aim (A) is reached when for each need, identified through qualitative analyses of interviews, one or more adjustments to the content or procedure of DELTA-JU is documented. No quantitative hypothesis is applicable. Aims (B) and (C) are achieved through testing the following hypotheses in adolescents who are abstinent for alcohol and drugs except nicotine/tobacco:

1. Adolescents: relevant increase in SUD-related subjective utility through FU (descriptive: a medium rating of 2.0 or higher in the respective GEJ items across participants);
2. Adolescents: significant reduction of substance-related craving as a measure of relapse risk (*p* < 0.05, MaCS score) through FU;
3. Adolescents: significant reduction of SUD severity (*p* < 0.05, scores of FTND, AUDIT, and DUDIT) through FU;
4. Adolescents: significant reduction of self-reported past-month tobacco use through FU (*p* < 0.05, substance use interview);
5. Personnel: relevant increase in SUD-related subjective utility through FU (descriptive: a medium rating of 2.0 or higher in the respective GEB items across participating personnel); and
6. Personnel: high satisfaction ratings concerning the workshop organization (descriptive: a medium rating of 4.0 or higher in the respective GEB items across participating personnel).

Additionally, secondary outcomes in adolescents (BDI-II, PQ-16, UCLA-PTSD, PSS-10, and SWLS) and personnel (ERI-S-10) will be tested exploratorily for changes through FU as previously done in the DELTA evaluation (15). All reductions through FU in adolescents are expected to be descriptively larger than reductions over the natural course, i.e., from PRE to BL in the WL group.

#### Estimation of sample size

Sample size will be optimized to achieve sufficient power for hypothesis testing. Assuming an error probability of α = 0.05, test power of 1−β = 0.95, a large effect of time with *f* = 0.40, and a repeated measures correlation of *r* = 0.1, we computed minimum sample sizes of *N* = 40 analyzed adolescents and *N* = 40 analyzed personnel with the G*Power software (64). Large effect sizes were anticipated given that the actual intervention effect size (15) had not yet been analyzed at the time of the inception of DELTA-JU and the proposal for funding and ethics approval in 2020.

Considering the dropout rates from BL to FU for adolescents (assuming 20%) and personnel (assuming 10%), at least *N* = 50 adolescents and *N* = 45 personnel will have to be included at BL. In case that dropout rates exceed 20%, we will continue to sample participants in order to achieve the required analysis sample size of *N* = 40 nonetheless as long as study funding is available. For qualitative interviews, a sample size of *N* = 10 was deemed as an adequate compromise between resources and expected output.

## Discussion

The DELTA-JU study will create a setting-specific manual for highly vulnerable adolescents who suffer from one or more SUDs, and, in many cases, from co-occurring mental disorders. We believe that DELTA-JU will be (a) properly adjusted to setting-specific needs of youth welfare institutions offering housing for adolescents with SUD, (b) accepted both by adolescents and the institutions’ personnel, and (c) effective in remaining abstinent from substances, reducing tobacco smoking, and reducing SUD problems. Providing feasible and effective treatments that are accepted by their respective target population is one primary goal set both for mental health research and practice in Europe (65).

As in all interventions, an age-specific approach is warranted when approaching children and adolescents. DELTA and DELTA-JU indeed provide an age-adequate intervention that includes material, methods, and content created for adolescents’ needs specifically while avoiding topics and approaches better suited for adults or older adults, e.g. (66). Furthermore, DELTA and DELTA-JU integrate modules on prevalent and outcome-relevant problems besides SUD, i.e., for depressiveness, for emotion regulation, and coping with stress and boredom. Addressing such co-occurring psychopathologies is in line with current recommendations for state-of-the art psychotherapeutic treatments (67). In the future, psychotherapeutic interventions such as DELTA-JU may be accompanied by including virtual reality elements or mobile phone-based elements.

Giving that effects can be independently replicated, it can be disseminated within other institutions of youth welfare. This will help institutions to professionalize their service for adolescents with SUD and it provides another currently lacking tool (68) for the outpatient treatment of SUDs before and after inpatient detoxification treatment (69). At the same time, we believe that besides treatment for diagnosed SUDs, efforts in prevention and early intervention are necessary nonetheless (70).

From a long-term perspective, programs such as DELTA-JU may help to alleviate the immense societal costs relating to consequences of alcohol use (27pprox. 32.5 billion EUR per year in Germany) (71), tobacco use (97.2 billion EUR) (72), cannabis use (0.9 billion EUR) (73), and the use of further substances for which no estimations are available.

### Strengths and limitations

The presented study design has several strengths. First, it is in line with ethical requirements that all adolescents are offered treatment. Waiting for 16 weeks in the WL condition still seems adequate given that adolescents may reside for up to 2 years in youth welfare institutions. In randomized clinical trials, for example, only cases may have received treatments. Second, it requires a smaller sample when participants become part of several analysis groups (intervention vs. WL). Third, alternative explanations of time effects may be controlled for by comparing BL-FU effects with PRE-BL effects.

Challenges arise from uncontrolled parallel treatments during the waiting period. Such treatments cannot be included in the quantitative estimation of treatment effects due to the limited number of participants in each group. Furthermore, homogeneity of cooperating youth welfare institutions is limited. Results may thus have to be replicated in other and diverging institutions. Although the sample size was *a-priori* calculated to maximize the use of available resources, drop-out rates may be unexpectedly high. In this case, additional participants and institutions may have to be recruited. In case of limited sample size, using Bayesan methods instead of testing for differences from zero may be advisable (74). Both the AUDIT and DUDIT have been previously used to assess changes in alcohol- and drug-related problems in adolescents and young adults using 3-month intervals between BL and FU (75, 76). Nevertheless, it remains possible that small changes in problems might go unnoticed given that both instruments cover 12 month periods. Another limitation arising from the limited sample size in a phase II study is the impossibility to adjust for several baseline variables. Such superior analysis strategies are not feasible at this point of the intervention development but will be imperative during phases III/RCT and phase IV studies in the future. The same holds true for the analysis of relevant patient subgroups (e.g., differentiating those with primarily alcohol-related problems from those with primarily cannabis-related problems) and intention-to-treat analyses or sensitivity analysis. Finally, additional measures that would objectify relapse to the use of alcohol or other drugs were not funded and will have to be implemented in a future trial, preferably a multi-center RCT testing DELTA-JU against treatment-as-usual.

## Conclusion

By developing the existing DELTA manual further in terms of setting-specific adaptions (DELTA-JU) for vulnerable adolescents suffering from SUDs, we may add another treatment option for this at-risk population. This requires evidence for acceptability, feasibility, and effectiveness as far as this exploratory trial is able to produce them. We hope that DELTA-JU can finally be disseminated within other institutions of youth welfare to help these adolescents to remain abstinent from alcohol and other drugs in the future.

## Ethics statement

This trial and all its procedures are in accordance with the Declaration of Helsinki. It has been submitted as an amendment to the DELTA evaluation study to the ethics committee of the University Hospital C. G. Carus Dresden and Technische Universität Dresden (approval in Jan 2022, number: EK 66022018 including amendments). It was furthermore pre-registered at the German Clinical Trials Register (DRKS, reference number: DRKS00027913, see www.drks.de/DRKS00027913), official as of 01.Feb 2022) where important protocol modifications will be communicated, see also SPIRIT 2013 checklist [75] (Supplementary information 1) and World Health Organization Trial Registration Data Set (Supplementary information 2) for details. An English version will be automatically uploaded to http://apps.who.int/trialsearch/. Both adolescent participants and personnel will have to consent to participate. In the case of adolescents aged 17 or younger, legal guardians as well have to agree to study participation by written consent after a comprehensive written and/or verbal information. Similar written information will be handed out to all participants, including information on study aims, procedures, duration, data security, voluntariness of participation and the right to leave the study at all times. Importantly, participants will be informed that any information on potentially illegal actions such as purchase, possession, sale, or use of illicit substances will not be forwarded to legal authorities, legal guardians or youth welfare institution personnel in accordance with data protection laws. Anonymity of study data will be assured by saving all information under an individual arbitrary ID code, with the linkage file between ID code and name only available to study personnel. All personnel including student assistants and master/doctoral candidates will undergo trainings on data security measures and good scientific practice as supervised by the principal investigators and/or the sponsoring institution. Study data will be hosted on servers of the funding institution (Technische Universität Dresden) in accordance with local data security laws. We do not seek to publish any identifying images or other personal or clinical details of adolescent participants that compromise anonymity. As for qualitative interviews of youth welfare institution personnel, we plan to publish anonymized sentences. Personnel will be informed in advance during the comprehensive written and/or verbal information, and will have to give written consent that they agree.

## Author contributions

SK-P conceived the study. SK-P, LB, VR, and YG contributed to the study design. LB contributed to the determination of the research directions for the DELTA manual. SK-P, VR, and YG are grant holders. YG is conducting the primary statistical analyses. SK-P conducted the additional analyses. YG will act as the leading principal investigator, responsible for preparation of protocol and revisions, preparation of study material and assessment procedures, recruitment of cooperating youth welfare institutions, randomization, data management, budget administration, supervision of study personnel, and conducting main analyses. YG will be part of the steering committee that also includes co-PIs SK-P and VR. This committee will meet regularly to support YG and to discuss issues regarding data monitoring, analyses, and publication of results with accordance to ICMJE guidelines for authorship eligibility (http://www.icmje.org/recommendations/). All authors contributed to the article and approved the submitted version.

## Funding

The Article Processing Charge (APC) were funded by the joint publication funds of the TU Dresden, including Carl Gustav Carus Faculty of Medicine, and the SLUB Dresden as well as the Open Access Publication Funding of the DFG. This work is externally funded by the public charitable foundation “Roland Ernst Stiftung für Gesundheitswesen” (https://roland-ernst-stiftung.com/; grant “DELTA-JU 6/21” to SK-P, VR, and YG), which provides funding for study personnel, participant reimbursements, breathalyzer etc. Additionally, the trial sponsor institution (Technische Universität Dresden, Faculty of Medicine, Department of Child and Adolescent Psychiatry) will provide working places, statistical software etc. Commercial funding was neither sought nor granted nor is it used for the study. The funding source had no role in the design of this study and will not have any role during its execution, analyses, interpretation of the data, or decision to submit results.

## Conflict of interest

SK-P, LB, VR, and YG are authors of the DELTA treatment manual, thus they receive honoraria for the manual as published by from Hogrefe Publishing.

## Publisher’s note

All claims expressed in this article are solely those of the authors and do not necessarily represent those of their affiliated organizations, or those of the publisher, the editors and the reviewers. Any product that may be evaluated in this article, or claim that may be made by its manufacturer, is not guaranteed or endorsed by the publisher.

## Abbreviations

BL/FU/PRE: Assessments during the study at baseline, follow-up or previous to baseline
DRKS: German Clinical Trials Register
DELTA: Dresden multimodal therapy for adolescents with chronic substance use (German abbreviation)
DELTA-JU: The revised DELTA program for youth welfare institutions (JU = in Jugendwohneinrichtungen in German)
ICMJE: International Committee of Medical Journal Editors
ID: Personal identification number used to anonymize study data
MANOVA: Multivariate analysis of variance
SPIRIT: Standard protocol items: recommendations for interventional trials
SUD: Substance use disorders
WL: Waiting list control group (study arm/condition)
