# Supplementary material for: Study protocol: the pragmatic, exploratory DELTA-JU trial of the group-based multimodal DELTA intervention for abstinent adolescents with substance use disorders living in youth welfare institutions
Source: Front Psychiatry. 2023 May 26;14:1025347. doi: 10.3389/fpsyt.2023.1025347 (PMC10298162; doi:10.3389/fpsyt.2023.1025347)
Supplement: Supplementary file 2 [file Data_Sheet_2.doc]

# World Health Organization Trial Registration Data Set

(Version 1.3.1, retrieved from <https://www.who.int/clinical-trials-registry-platform/network/who-data-set> at 19.Jan 2022)

| Item No | Item | Description |  |
| --- | --- | --- | --- |
| 1 | **Primary Registry and Trial Identifying Number** | Name of Primary Registry, and the unique ID number assigned by the Primary Registry to this trial. | 🡪 German Clinical Trials Register (DRKS), ID number: DRKS00027913 |
| 2 | **Date of Registration in Primary Registry** | Date when trial was officially registered in the Primary Registry. | 01. Feb 2022 |
| 3 | **Secondary Identifying Numbers** | Other identifiers besides the Trial Identifying Number allocated by the Primary Registry, if any. These include:   - The Universal Trial Number (UTN) - Identifiers assigned by the sponsor (record Sponsor name and Sponsor-issued trial number (e.g. protocol number)) - Other trial registration numbers issued by other Registries (both Primary and Partner Registries in the WHO Registry Network, and other registries) - Identifiers issued by funding bodies, collaborative research groups, regulatory authorities, ethics committees / institutional review boards, etc. - All secondary identifiers will have 2 elements: an identifier for the issuing authority (e.g. NCT, ISRCTN, ACTRN) plus a number. | Private sponsorship: Roland Ernst Stiftung für Gesundheitswesen „DELTA-JU 6/21“ |
| 4 | **Source(s) of Monetary or Material Support** | Major source(s) of monetary or material support for the trial (e.g. funding agency, foundation, company, institution). | Technische Universität Dresden, Faculty of Medicine, Dept. of Child and Adolescent Psychiatry |
| 5 | **Primary Sponsor** | The individual, organization, group or other legal entity which takes responsibility for initiating, managing and/or financing a study. The Primary Sponsor is responsible for ensuring that the trial is properly registered. The Primary Sponsor may or may not be the main funder. | Technische Universität Dresden, Faculty of Medicine, Dept. of Child and Adolescent Psychiatry |
| 6 | **Secondary Sponsor(s)** | Additional individuals, organizations or other legal persons, if any, that have agreed with the primary sponsor to take on responsibilities of sponsorship. A secondary sponsor may have agreed to:   - take on all the responsibilities of sponsorship jointly with the primary sponsor; or - form a group with the Primary Sponsor in which the responsibilities of sponsorship are allocated among the members of the group; or - act as the Primary Sponsor’s legal representative in relation to some or all of the trial sites. | Not applicable. |
| 7 | **Contact for Public Queries** | Email address, telephone number and postal address of the contact who will respond to general queries, including information about current recruitment status. *“Note: The information provided in here is functional and not personal, it is recommended to provide institutional and not personal information. By providing this information the registrant consents that the information provided can or may be published on a public website. Once provided the information cannot be redacted or anonymized as a result of new privacy legislation such as the European General Data Protection Regulation (GDPR)”.* | - [KJPForschung@uniklinikum-dresden.de](mailto:KJPForschung@uniklinikum-dresden.de), - +49-351-458-7168 - Fetscherstr 74, 01307 Dresden, Germany |
| 8 | **Contact for Scientific Queries** | There must be clearly assigned responsibility for scientific leadership to a named Principal Investigator. The PI may delegate responsibility for dealing with scientific enquiries to a scientific contact for the trial. This scientific contact will be listed in addition to the PI.  *“Note: The information provided in here is functional and not personal, it is recommended to provide institutional and not personal information. By providing this information the registrant consents that the information provided can or may be published on a public website. Once provided the information cannot be redacted or anonymized as a result of new privacy legislation such as the European General Data Protection Regulation (GDPR)”.*  The contact for scientific queries must include:   - Name and title, email address, telephone number, postal address and affiliation of the Principal Investigator, and; - Email address, telephone number, postal address and affiliation of the contact for scientific queries about the trial (if applicable). The details for the scientific contact may be generic (that is, there does not need to be a named individual): e.g. a generic email address for research team members qualified to answer scientific queries. | PD Dr. Dr. Yulia Golub:   - [Yulia.golub@ukdd.de](mailto:Yulia.golub@ukdd.de) - +49-351-458-2282 - Fetscherstr 74, 01307 Dresden, Germany |
| 9 | **Public Title** | Title intended for the lay public in easily understood language. | Anpassung und Bewertung des DELTA-Behandlungsprogramms für Jugendliche mit Suchterkrankungen in Jugendwohngruppen (adaption and evaluation of the DELTA treatment for adoelscents with addictions in youth welfare homes) |
| 10 | **Scientific Title** | Scientific title of the study as it appears in the protocol submitted for funding and ethical review. Include trial acronym if available. | Evaluierung des DELTA-Behandlungsprogramms für Jugendliche mit Substanzkonsumstörung nach Anpassung für vollstationäre Einrichtungen der Jugendhilfe (evaluation of the DELTA treatment program for adolescents with substance use disorder, adapted for youth welfare homes) |
| 11 | **Countries of Recruitment** | The countries from which participants will be, are intended to be, or have been recruited at the time of registration. | Germany |
| 12 | **Health Condition(s) or Problem(s) Studied** | Primary health condition(s) or problem(s) studied (e.g., depression, breast cancer, medication error). If the study is conducted in healthy human volunteers belonging to the target population of the intervention (e.g. preventive or screening interventions), enter the particular health condition(s) or problem(s) being prevented. | Substance use disorders, substance abuse, drug addiction |
| 13 | **Intervention(s)** | For each arm of the trial record a brief intervention name plus an intervention description. Intervention Name: For drugs use generic name; for other types of interventions provide a brief descriptive name.   - For investigational new drugs that do not yet have a generic name, a chemical name, company code or serial number may be used on a temporary basis. As soon as the generic name has been established, update the associated registered records accordingly. - For non-drug intervention types, provide an intervention name with sufficient detail so that it can be distinguished from other similar interventions.   Intervention Description: Must be sufficiently detailed for it to be possible to distinguish between the arms of a study (e.g. comparison of different dosages of drug) and/or among similar interventions (e.g. comparison of multiple implantable cardiac defibrillators). For example, interventions involving drugs may include dosage form, dosage, frequency and duration. | Immediate condition: receives DELTA-JU treatment after initial assessment  Waitlist condition: receives DELTA-JU treatment 16 weeks after initial assessment |
| 14 | **Key Inclusion and Exclusion Criteria** | Inclusion and exclusion criteria for participant selection, including age and sex. Other selection criteria may relate to clinical diagnosis and co-morbid conditions; exclusion criteria are often used to ensure patient safety. If the study is conducted in healthy human volunteers not belonging to the target population (e.g. a preliminary safety study), enter "healthy human volunteer". | Inclusion of adolescent participants:   - aged 12.00 – 17.99 years - SUD diagnosis or chronic substance use during the past   Exclusion of adolescent participants:   - intelligence quotient is below 70 - did not attend any of the 16 sessions for whatever reason (applies only to BL-FU analyses) - study participation was discontinued due to an adverse event (applies only to BL-FU analyses)   Inclusion of personnel of youth welfare institutions   - if they work with at least 20h/week in a cooperating institution |
| 15 | **Study Type** | Study type consists of:   - Type of study (interventional or observational) - Study design including:   - Method of allocation (randomized/non-randomized)   - Masking (is masking used and, if so, who is masked)   - Assignment (single arm, parallel, crossover or factorial)   - Purpose - Phase (if applicable)   For randomized trials: the allocation concealment mechanism and sequence generation will be documented. | - Type: Interventional - Allocation: Cluster-randomized, non-masked / unblended, two parallel arms - Phase: Phase II |
| 16 | **Date of First Enrollment** | Anticipated or actual date of enrolment of the first participant. | Actual: 02. Feb 2022 |
| 17 | **Sample Size** | Sample Size consists of:   - Number of participants that the trial plans to enrol in total. - Number of participants that the trial has enrolled | Planned:   - *N*=50 adolescents - *N*=45 personnel - *N*=10 personnel for qualitative interviews   Currently enrolled: not applicable |
| 18 | **Recruitment Status** | Recruitment status of this trial:   - Pending: participants are not yet being recruited or enrolled at any site - Recruiting: participants are currently being recruited and enrolled - Suspended: there is a temporary halt in recruitment and enrolment - Complete: participants are no longer being recruited or enrolled - Other | Pending |
| 19 | **Primary Outcome(s)** | Outcomes are events, variables, or experiences that are measured because it is believed that they may be influenced by the intervention.  The Primary Outcome should be the outcome used in sample size calculations, or the main outcome(s) used to determine the effects of the intervention(s). Most trials should have only one primary outcome.  For each primary outcome provide:   - The name of the outcome (do not use abbreviations) - The metric or method of measurement used (be as specific as possible) - The timepoint(s) of primary interest   Example: Outcome Name: Depression Metric/method of measurement: Beck Depression Score Timepoint: 18 weeks following end of treatment | Outcome name: SUD severity  Method of measurement: scores from AUDIT, DUDIT, FTND  Timepoints: baseline, 16-week follow-up  Outcome name: Craving  Method of measurement: MaCS score  Timepoints: baseline, 16-week follow-up  Outcome name: Substance use  Method of measurement: past-month quantity & frequency reported in generic interview  Timepoints: baseline, 16-week follow-up  Outcome name: SUD-related knowledge  Method of measurement: scoring of items in generic questionnaires (GEJ, GEB)  Timepoints: baseline, 16-week follow-up |
| 20 | **Key Secondary Outcomes** | Secondary outcomes are outcomes which are of secondary interest or that are measured at timepoints of secondary interest. A secondary outcome may involve the same event, variable, or experience as the primary outcome, but measured at timepoints other than those of primary interest.  As for primary outcomes, for each secondary outcome provide:   - The name of the outcome (do not use abbreviations) - The metric or method of measurement used (be as specific as possible) - The timepoint(s) of interest | Outcome name: Depressiveness  Method of measurement: BDI-II score  Timepoints: baseline, 16-week follow-up  Outcome name: Psychopathologies  Method of measurement: YSR subscales  Timepoints: baseline, 16-week follow-up  Outcome name: post-traumatic stress symptoms  Method of measurement: UCLA PTSD scale scores  Timepoints: baseline, 16-week follow-up  Outcome name: Prodromal psychotic symptoms  Method of measurement: PQ-16 score  Timepoints: baseline, 16-week follow-up  Outcome name: Satisfaction with life  Method of measurement: SWLS score  Timepoints: baseline, 16-week follow-up  Outcome name: Perceived past-month stress  Method of measurement: PSS-10 score, ERI-S-10 score  Timepoints: baseline, 16-week follow-up |
| 21 | **Ethics Review** | The ethics review process information of the trial record in the primary register database. It consists of:   - Status (possible values: Not approved, Approved, Not Available) - Date of approval - Name and contact details of Ethics committee(s) | Approved (07. Jan 2022) by the Institutional Review Board / ethics committee of the University Hospital C. G. Carus Dresden (EK 66022018 including amendments). |
| 22 | **Completion date** | Date of study completion: The date on which the final data for a clinical study were collected (commonly referred to as, "last subject, last visit"). | Approx. Dez 2023 |
| 23 | **Summary Results** | It consists of:   - Date of posting of results summaries - Date of the first journal publication of results - URL hyperlink(s) related to results and publications - Baseline Characteristics: Data collected at the beginning of a clinical study for all participants and for each arm or comparison group. These data include demographics, such as age and sex, and study-specific measures. - Participant flow: Information to document the progress and numbers of research participants through each stage of a study in a flow diagram or tabular format. - Adverse events: An unfavorable change in the health of a participant, including abnormal laboratory findings, and all serious adverse events and deaths that happen during a clinical study or within a certain time period after the study has ended. This change may or may not be caused by the intervention being studied. - Outcome measures: A table of data for each primary and secondary outcome measure and their respective measurement of precision (eg a 95% confidence interval) by arm (that is, initial assignment of participants to arms or groups) or comparison group (that is, analysis groups), including the result(s) of scientifically appropriate statistical analyses that were performed on the outcome measure data, if any. - URL link to protocol file(s) with version and date - Brief summary | Not yet applicable. |
| 24 | **IPD sharing statement** | Statement regarding the intended sharing of deidentified individual clinical trial participant-level data (IPD). Should indicate whether or not IPD will be shared, what IPD will be shared, when, by what mechanism, with whom and for what types of analyses. It consists of:   - Plan to share IPD (Yes, No) - Plan description | IPD will not be shared. |
